# The complete mitochondrial genomes of *Bothrogonia tongmaiana* and *Bothrogonia yunana* (Hemiptera: Cicadellidae) from China

**DOI:** 10.1080/23802359.2021.1875910

**Published:** 2021-02-19

**Authors:** Xiao-Li Xu, Bin Yan, Xiao-Fei Yu, Mao-Fa Yang

**Affiliations:** a College of Agriculture, Guizhou University, Guiyang, PR China; b Guizhou Provincial Key Laboratory for Agricultural Pest Management of the Mountainous Region, Institute of Entomology, Guizhou University, Guiyang, PR China; c College of Tobacco Science, Guizhou University, Guiyang, PR China; d Guizhou Key Laboratory of Tobacco Quality Research, Guiyang, PR China

**Keywords:** Mitochondrial genome, *Bothrogonia tongmaiana*, *Bothrogonia yunana*, phylogenetic analyses

## Abstract

The complete mitochondrial genomes of *Bothrogonia tongmaiana* and *Bothrogonia yunana* are sequenced and annotated, which are 15,539 and 15,585 bp in length, respectively. *Bothrogonia tongmaiana* has an A + T content of 79.4% (A 55.7%; C 13.5%; G 7.1%, and T 23.7%), while *B. yunana* has an A + T content of 80.6% (A 37.5%; C 5.6%; G 13.8%, and T 43.1%). Every mitogenome encodes 13 proteins, 2 ribosomal RNAs, 22 tRNAs, and a control region. The phylogenetic tree was reconstructed based on them and 27 reference species, which could further confirm the status of these two species.

The genus *Bothrogonia* China is widely distributed in the world and is known to be distributed in the Palearctic, Oriental, and Afrotropical Realm. Forty-seven species have been recorded worldwide, while 38 species have been recorded in China. The model specimens of *Bothrogonia tongmaiana* and *Bothrogonia yunana* are located in Xizang and Yunnan, China (China 1938; Yang and Li 1980; Yang et al. 2017). In this study, the complete mitogenomes of them were present sequenced and annotated, which would be a significant increase in further study on phylogenetic analyses of the subfamily Cicadellidae.

Both of *B. tongmaiana* and *B. yunana* were collected from Baoshan City, Yunnan Province, China. The former was at an elevation of 1800 m in the Linjiapu Forest Farm, and the latter was at an altitude of 1200 m in Gaoligong Mountain National Nature Reserve. Total DNA was extracted from a male adult. Every genitalia is deposited in the Institute of Entomology, Guizhou University, Guiyang, China (GUGC), and the deposited numbers are GUGC-IDT-00202 and GUGC-IDT-00203 (Zhang 2018). The related experiments were carried out by NIKON NI-E + DS-RI2 + NIS-AR instrument. The high-throughput sequencing method was used to obtain the complete mitochondrial genomes by Illumina NovaSeq6000 platform (Berry Genomics, Beijing, China). The raw data were assembled and annotated by NOVOPlasty, MitoZ, and Generous Prime (Dierckxsens et al. 2017; Meng et al. 2019). Annotated sequences were submitted to Genbank with accession number MT500857 for *B. tongmaiana* and MT500858 for *B. yunana*. The phylogenetic tree (Figure 1), using the partition model determined by maximum likelihood method in IQ-TREE software, was reconstructed based on amino acid sequences of 13 protein-coding genes (PCGs) by 29 species (Zhong et al. 2019, 2020). The tree was visualized in FigTree version 1.4.2 and edited using Adobe Illustrator CC 2018 (Rambaut 2016).

**Figure 1.**
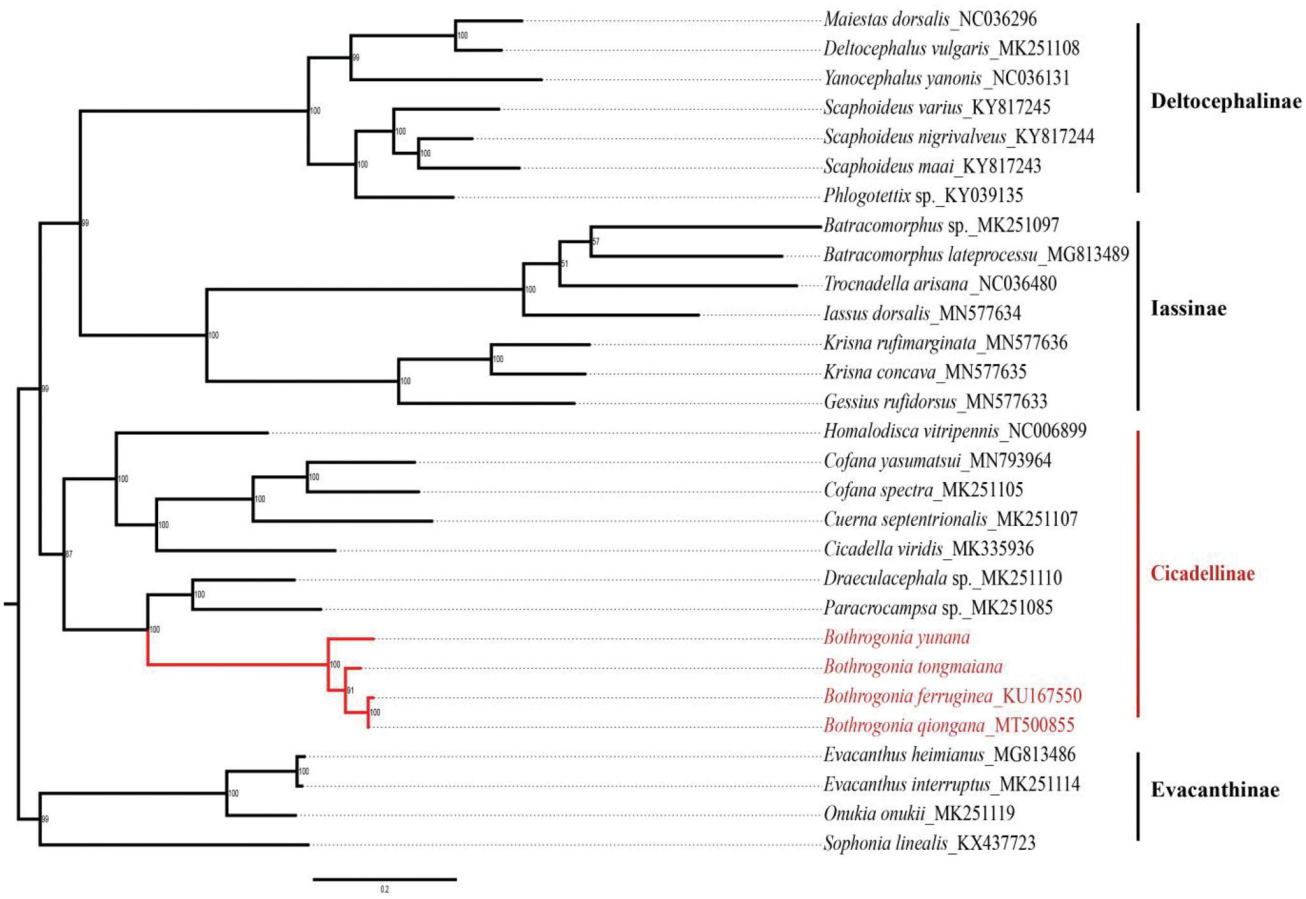
Phylogenetic analyses of *Bothrogonia tongmaiana* and *Bothrogonia yunana* based on the amino acid sequences of the 13 PCGs. (Numbers at nodes are bootstrap values. The GenBank accession number for each species is indicated after the scientific name.)

The complete mitogenomes of *B. tongmaiana* and *B. yunana* are 15,539 and 15,585 bp in length, respectively. Every mitogenome contains 13 PCGs, 22 transfer RNA genes (tRNA), 2 ribosomal RNA genes (rRNA), and a contol region (C-region). *Bothrogonia tongmaiana* has an A + T content of 79.4% (A 55.7%; C 13.5%; G 7.1%, and T 23.7%), while *B. yunana* has an A + T content of 80.6% (A 37.5%; C 5.6%; G 13.8%, and T 43.1%). All PCGs start with ATN (*ATA*, *ATC*, *ATT*, and *ATG*) expect ND5s which start with TTG. Most of the PCGs used typical TAA or TAG as stop codon, but both COX2 end with incomplete single T and ND1 of the latter terminated also. All the tRNAs are between 59 bp (tRNA-R, *B. yunana*) and 73 bp (tRNA-M, *B. yunana)* in size. The length of 16S rRNA and 12S rRNA gene is 1158 and 817 bp in *B. tongmaiana*, and another one has 1165 and 784 bp, respectively. The 1188 and 1258 bp control regions of *B. tongmaiana* and *B. yunana* were located between 12S rRNA and RNA-I.

The tree further confirmed that *B. tongmaiana* and *B. yunana* are closely related to *B. ferruginea* and *B. qiongana.* They are clustered into one clade, which is congruent with traditional taxonomy. As a result, mitogenome data could provide more information on population genetics and evolution of Cicadellidae.

## Data Availability

The data that support the findings of this study are openly available in GenBank of NCBI at https://www.ncbi.nlm.nih.gov, reference number MT500857 and MT500858.
